# Modern contraceptive availability and stockouts: a multi-country analysis of trends in supply and consumption

**DOI:** 10.1093/heapol/czaa197

**Published:** 2021-01-17

**Authors:** Pierre Muhoza, Alain K Koffi, Philip Anglewicz, Peter Gichangi, Georges Guiella, Funmilola OlaOlorun, Elizabeth Omoluabi, P R Sodani, Mary Thiongo, Pierre Akilimali, Amy Tsui, Scott Radloff

**Affiliations:** Department of International Health, Johns Hopkins Bloomberg School of Public Health, 615 N. Wolfe Street, Baltimore, MD 21205, USA; Department of International Health, Johns Hopkins Bloomberg School of Public Health, 615 N. Wolfe Street, Baltimore, MD 21205, USA; Department of Population, Family and Reproductive Health, Johns Hopkins Bloomberg School of Public Health, 615 N. Wolfe Street, Baltimore, MD 21205, USA; International Centre for Reproductive Health Kenya, Nairobi, Kenya; Institut Supérieur des Sciences de la Population (ISSP) of the Joseph Ki-Zerbo University, Ouagadougou, Burkina Faso; Department of Community Medicine, University of Ibadan, Ibadan, Nigeria; Centre for Research, Evaluation Resources and Development, Ife, Nigeria; IIHMR University, Jaipur, India; International Centre for Reproductive Health Kenya, Nairobi, Kenya; Ecole de Santé Publique de l'Université de Kinshasa, Kinshasa, Democratic Republic of Congo; Department of Population, Family and Reproductive Health, Johns Hopkins Bloomberg School of Public Health, 615 N. Wolfe Street, Baltimore, MD 21205, USA; Department of Population, Family and Reproductive Health, Johns Hopkins Bloomberg School of Public Health, 615 N. Wolfe Street, Baltimore, MD 21205, USA

**Keywords:** Family planning, contraception, reproductive health, survey methods, surveillance, public/private, health services

## Abstract

Approximately 214 million women of reproductive age lack adequate access to contraception for their family planning needs, yet patterns of contraceptive availability have seldom been examined. With growing demand for contraceptives in some areas, low contraceptive method availability and stockouts are thought to be major drivers of unmet need among women of reproductive age, though evidence for this is limited. In this research, we examined trends in stockouts, method availability and consumption of specific contraceptive methods in urban areas of four sub-Saharan African countries (Burkina Faso, Democratic Republic of Congo, Kenya and Nigeria) and India. We used representative survey data from the Performance Monitoring for Action Agile Project that were collected in quarterly intervals at service delivery points (SDP) stratified by sector (public vs private), with all countries having five to six quarters of surveys between 2017 and 2019. Among SDPs that offer family planning, we calculated the percentage offering at least one type of modern contraceptive method (MCM) for each country and quarter, and by sector. We examined trends in the percentage of SDPs with stockouts and which currently offer condoms, emergency contraception, oral pills, injectables, intrauterine devices and implants. We also examined trends of client visits for specific methods and the resulting estimated protection from pregnancy by quarter and country. Across all countries, the vast majority of SDPs had at least one type of MCM in-stock during the study period. We find that the frequency of stockouts varies by method and sector and is much more dynamic than previously thought. While the availability and distribution of long-acting reversible contraceptives (LARCs) were limited compared to other methods across countries, LARCs nonetheless consistently accounted for a larger portion of couple years of protection. We discuss findings that show the importance of engaging the private sector towards achieving global and national family planning goals.

**KEY MESSAGES**

- PMA Agile is a useful platform for routinely tracking the family planning supply environment in urban/suburban areas of low- and middle-income countries
- Changes in contraceptive supply are common and vary by method and sector (public vs private)
- Pharmacies and drug shops could be further leveraged in addressing unmet need in contraceptives

## Introduction

Providing access to contraception is critical for achieving several global health outcomes, such as reducing maternal mortality, maximizing the health benefits of birth spacing, and promoting the economic empowerment of women (Stover and Ross, 2010; Ahmed*et al.*, 2012; Do and Kurimoto, 2012). Considering the efforts of family planning (FP) programs worldwide to address the unmet need for contraception, progress has been slow in many low-resource countries (Darroch *et al.*, 2011). Millions of women continue to lack access to contraception due to ‘supply-side’ barriers such as poor accessibility to health facilities, low levels of contraceptive method availability and contraceptive stockouts (Hubacher *et al.*, 2008; Chandra-Mouli*et al.*, 2014).

In 2017, more than 214 million women of reproductive age worldwide lacked adequate access to contraception (Guttmacher Institute, 2017). Stockouts likely play a role in this lack of access, though the relationship between contraceptive availability and use remains poorly understood. Existing evidence suggests that contraceptive stockouts and method availability range widely across countries and across methods (Ali *et al.*, 2018; Babazadeh *et al.*, 2018; Zimmerman *et al.*, 2019). Different countries may experience different contexts of varying supply chain challenges that ultimately impact method availability and stockouts differentially. The policy environment on family planning also likely contributes to the variation in contraceptive stockouts and method availability across health delivery sectors. For instance, a 2018 USAID report suggested that among the 36 countries providing information on supply chain challenges, 15% cited formal and informal policy barriers that hinder the ability of the private sector to provide contraceptive methods (USAID, 2018).

Stockouts and low method availability restrict choice in contraception, forcing individuals to choose methods that may not suit their preferences and needs. For example, a woman’s contraceptive preferences may vary depending on spousal preferences, cultural acceptability of contraceptive methods, socioeconomic influences, her motivation for spacing or limiting births and her preference for hormonal or non-hormonal methods (Brown and Eisenberg, 1995; Wang and Mallick, 2019). In limiting contraceptive method choice, stockouts and low method availability promote conditions that ultimately discourage the use of modern methods and likely lead to increased contraceptive discontinuation (Ross and Hardee, 2013; Grindlay *et al.*, 2016). Therefore, ensuring an adequate range of methods at various levels of the health care system is crucial to guarantee that individuals and couples can select their contraceptive method of choice, thereby allowing them to achieve their fertility goals.

Despite the importance of measuring stockouts, existing research is limited. Data on the family planning supply side are not systematically available: the Service Provision Assessment (SPA) of the Demographic and Health Surveys (DHS) occurs at irregular intervals and only in a limited set of countries where DHS operates (DHS Program, 2017). Overall, data on stockouts by contraceptive method are scarce, and only a few countries are able to monitor stockouts routinely at the facility level (FP, 2015, 2020). Furthermore, since there have not been many studies that have examined contraceptive stockouts in the context of client volume, it remains unclear whether stockouts are primarily a response to a break-down in the supply chain or increase in demand, or both. It is plausible that stockouts may be broadly problematic for the most popularly used methods, especially short-acting methods that require frequent revisits to maintain protection against unintended pregnancy. Due to these data limitations, little is known about patterns in stockouts, or the FP supply-side picture overall.

Data from the PMA Agile surveys provide an opportunity to address existing knowledge gaps on contraceptive stockouts. A unique feature of the PMA Agile platform is a design that allows the monitoring of progress at the subnational level of each country through the collection of facility-level data in quarterly intervals each year. They thus enable a continuous assessment of contraceptive commodity and service provision in urban facilities, both private and public.

In this research, we describe trends in stockouts, method availability and consumption of specific contraceptive methods using locally representative data from facilities in urban areas of Burkina Faso, the Democratic Republic of Congo (DRC), Kenya, Nigeria and India. We expect that this information may help monitor progress towards FP2020 goals (Brown *et al.*, 2014) and inform cross-country strategies to anticipate, reduce and prevent stockouts.

## Methods

### Study design, sampling and data collection

Data for this study come from the Performance Monitoring for Action (PMA) Agile Project. PMA Agile is a continuous data monitoring and evaluation system that collects data on family planning service delivery and consumption through quarterly public and private health facility surveys and semi-annual client exit interviews (CEIs) in urban areas. A phone follow-up survey is conducted with consenting female clients four months after their interviews (www.pmadata.org/technical-areas/pma-agile). It operates in selected urban areas of six countries, Burkina Faso, DRC, India, Kenya, Niger and Nigeria. PMA Agile has multiple sites in urban areas of four countries: Lagos, Kano and Ogun in Nigeria; Uasin Gishu, Migori and Kericho in Kenya; Indore, Firozabad and Puri in India; Ouagadougou and Koudougou in Burkina Faso. There is one PMA Agile site in each of the two remaining countries: Kinshasa in the DRC and Niamey in Niger. We note that the sites in Kenya are a mix of urban and suburban sites. Data from Niger are excluded from this study since they were not available at the time of this analysis. The population estimates for each of the aforementioned sites are included in the PMA Agile protocol paper (Tsui *et al.*, 2020).

The surveys in this study were implemented between November 2017 and December 2019 across countries. Within each country survey, data were collected by locally recruited and trained female resident enumerators (REs). Data collection was implemented using questionnaires powered by smartphone and open source software (Open Data Kit—ODK) technologies.

Our analysis included service delivery points (SDPs) data from official listings provided by the country Ministry of Health (MoH) and other government agencies within each PMA Agile study site. The sampling scheme allowed for a 10% non-response rate, and thus a maximum sample of 220 SDPs was randomly selected from each site. The surveys used a two-stage cluster sampling design where SDPs were first stratified by public and private and then further sampled based on probability proportional to size to select facility types with at least 20 SDPs. In smaller areas such as Koudougou where the number of SDPs was relatively limited, the full census of SDPs was used. More information about PMA Agile can be found at the project’s website: www.pmadata.org/technical-areas/pma-agile, and in the PMA Agile protocol paper (Tsui *et al.*, 2020).

### Measurement

The main measures of interest were the provision and demand of specific modern contraceptive methods (MCM) among SDPs offering family planning services. We define contraceptive stockouts as when one or more contraceptive methods are temporarily unavailable at a health facility that routinely provides that method (Grindlay *et al.*, 2016). Method availability, in turn, measures the percentage of health facilities offering a given contraceptive method over a period of time. Information on the country-specific brands of contraceptive methods considered is available on the project’s website and dashboard. The availability of family planning services/products at a given SDP was determined by the response to the survey question, ‘Do you usually offer FP services/products?’. If the SDP provided FP services/products, the respondent was then asked about the availability of specific contraceptive methods. If the SDP reported offering a given contraceptive method, the RE then asked if the method was in-stock on the day of the survey and if there had been a stockout of the method at any point within the 3 months preceding the interview. A method was considered in-stock only if the RE could visually confirm its availability on the day of the survey. To assess the demand of contraceptive methods, the RE requested to see the facility logbook and recorded the total number of family planning visits (new and continuing) in the last completed month for each method.

A secondary measure examined in this study was a couple years of protection (CYP). The CYP is a commonly used family planning metric that quantifies the level of protection offered by contraceptives against unintended pregnancies over a period of time (Stover and Ross, 2010). It is obtained by multiplying the quantity of each method distributed to clients by a conversion factor resulting in an estimate of the duration of contraceptive protection provided per unit of that method (USAID, 2019). To obtain the quantity of methods distributed to clients, we combined the total number of contraceptive method units sold with the total number of visits for each method. This was necessary since certain types of SDPs such as pharmacies, drugstores and chemists track product distribution as sales whereas other facility types use clinical visits as measures of client volume. To standardize these different measures, we assumed that clients received six condoms, four sachets of oral pills or one unit of the other methods for each relevant visit to an eligible facility.

We defined the following contraceptive methods as modern according to the WHO (WHO, 2018): oral pills, intrauterine devices (IUDs), injectables, male and female condoms, implants and emergency contraception. Though PMA Agile collects SDP data on other methods such as contraceptive beads, foam/jelly and sterilization, these are excluded from this study since the first two methods (beads, foam/jelly) are uncommon in all PMA Agile settings, and sterilization is a medical procedure for which stock does not directly apply as a measure. We note that PMA Agile did not collect information on implants in India as the method was not offered at the time of this study and is currently undergoing consideration for introduction into the national family planning program (Joshi *et al.*, 2019).

### Data analysis

We limited the analysis to SDPs offering family planning and stratified our analyses by sector (i.e. public vs private). The proportions of surveyed SDPs offering family planning for each country are shown in Supplementary Table S1. For each country, we first tabulated SDP types by quarter for those with stock of at least one type of modern contraceptive method. Though the standard in the field is to calculate the percentage of SDPs offering at least three to five methods in order to evaluate the level of client choice in methods, we found these thresholds to be too restrictive for analyses focused on assessing method stockouts. Next, we calculated the quarterly percentages of SDPs that typically offer specific contraceptive methods, those that had the indexed methods in stock, out-of-stock or had experienced a stockout within the 3-month period preceding the survey. We then estimated the total monthly volume of clients visiting SDPs for specific contraceptive methods. Finally, we estimated the percent contribution of individual contraceptive methods to overall CYP units produced.

Based on the sampling procedures, we constructed facility weights using SDP selection probabilities. The weighting process took into account the quarterly changes in non-response rates and any SDP classification change by public/private or facility type that happened over time. We report weighted results to account for the stratified two-stage cluster sampling design and the variances of the estimates are adjusted accordingly using Taylor series linearization. We used Stata version 14.2 (StataCorp LLC, College Station, TX) with the *SVY* command to conduct design‐based analyses that accounted for stratification, clustering and probability of selection of the SDPs (Heeringa *et al.*, 2017).

## Results

In Table 1, we present the number of SDPs by country and quarter, separately for public and private SDPs. We also show the percentage of each offering at least one family planning method in-stock, again by country, quarter and public/private.

**Table 1:** Percentage of SDPs (by type, country and quarter) with at least one type of modern contraceptive method in stock at time of survey

Country name	Quarters																							
	Public SDPs											Private SDPs												
	Q1		Q2		Q3		Q4		Q5		Q6		Q1		Q2		Q3		Q4		Q5		Q6	
	*N*	%	*N*	%	*N*	%	*N*	%	*N*	%			*N*	%	*N*	%	*N*	%	*N*	%	*N*	%		
Burkina Faso																								
Hospital	2	100.0	2	73.9	2	100.0	2	72.7	1	100.0			2	0.0	0	0.0	2	0.0	2	0.0	2	0.0		
Medical Center/ Health Clinic/ Health Center	27	100.0	26	98.0	27	100.0	30	93.3	24	95.3			49	69.5	44	68.7	51	63.9	43	72.5	45	72.8		
Maternity clinic	13	56.7	11	49.9	13	52.6	11	48.2	11	42.7			4	50.0	4	55.9	3	66.7	3	66.7	4	50.0		
Pharmacy	0	0.0	0	0.0	0	0.0	0	0.0	0	0.0			54	100.0	60	90.6	55	100.0	52	96.9	60	100.0		
All SDP types	42	86.9	39	82.7	41	84.5	43	81.4	36	78.9			109	83.0	108	80.6	111	81.2	100	83.8	111	85.7		
DRC																								
Hospital	13	81.8	19	93.8	16	92.9	17	100.0	17	78.6			1	100.0	1	100.0	1	100.0	1	100.0	1	100.0		
Health Center	64	78.9	54	93.5	60	90.5	59	96.0	60	94.1			11	90.0	11	100.0	11	100.0	12	81.8	12	100.0		
Pharmacy	0	0.0	0	0.0	0	0.0	0	0.0	0	0.0			58	92.9	62	93.2	54	98.5	54	93.6	53	95.2		
All SDP types	77	86.9	73	93.5	76	91.0	76	96.9	77	90.7			70	92.6	74	94.2	66	98.8	67	91.4	66	95.9		
India																								
Hospital	5	88.9	4	100.0	4	100.0	4	100.0	4	100.0	3	100.0	57	24.4	61	34.1	64	17.5	59	10.6	62	13.4	55	10.9
Health Clinic/Health Center	17	87.0	16	93.1	13	96.6	13	96.8	13	96.8	11	100.0	21	31.9	22	30.1	21	32.1	23	20.1	23	20.1	19	2.0
Pharmacy/Drugstore	0	0.0	0	0.0	0	0.0	0	0.0	0	0.0	0	0.0	217	94.0	224	96.8	216	96.1	217	78.0	216	78.8	168	68.6
Dispensary	4	100.0	3	100.0	4	100.0	3	100.0	3	100.0	3	100.0	0	0.0	0	0.0	0	0.0	0	0.0	0	0.0	0	0.0
All SDP types	25	89.3	23	95.4	21	97.8	20	97.9	20	97.9	17	100.0	295	76.1	307	79.6	301	74.9	299	60.1	301	61.0	242	50.2
Kenya																								
Hospital	14	100.0	14	95.5	14	90.9	14	100.0	14	90.9	14	90.3	15	84.0	16	90.2	15	95.9	16	100.0	14	100.0	15	100.0
Health Clinic/Health Center	34	95.8	33	100.0	33	98.1	33	96.1	33	97.9	35	92.1	63	94.7	66	97.0	64	96.4	65	95.8	67	93.0	66	94.8
Pharmacy	0	0.0	0	0.0	0	0.0	0	0.0	0	0.0	0	0.0	166	100.0	181	99.5	195	100.0	202	99.5	188	99.5	198	99.5
Dispensary	184	97.7	175	99.3	172	99.5	173	97.5	171	98.6	174	97.9	22	91.9	25	84.7	21	100.0	23	100.0	22	94.6	22	100.0
All SDP types	232	97.6	222	99.1	219	98.7	220	97.5	218	98.0	223	96.5	266	97.2	288	97.1	295	99.0	306	98.8	293	97.6	301	98.6
Nigeria																								
Hospital	5	94.9	6	100.0	6	95.0	6	95.1	5	95.0	5	100.0	133	70.5	164	78.8	169	69.9	168	59.5	151	56.9	145	63.9
Health Post/Health Center	75	89.3	83	92.8	83	91.7	83	91.6	77	94.3	74	91.2	30	26.7	43	56.3	34	84.1	34	88.5	30	85.6	30	93.4
Maternity clinic	0	0.0	0	0.0	0	0.0	0	0.0	0	0.0	0	0.0	23	63.0	29	63.9	31	78.7	30	53.2	28	63.7	27	53.5
Pharmacy/Chemist	0	0.0	0	0.0	0	0.0	0	0.0	0	0.0	0	0.0	138	96.5	168	99.7	180	99.5	180	97.1	164	99.0	158	99.0
All SDP types	80	89.7	89	93.3	89	91.9	89	91.8	82	94.3	79	91.8	324	76.9	404	84.0	414	84.6	412	77.9	373	78.3	360	81.0

*N* represents the total weighted number of SDPs offering family planning

Overall, some patterns in the health system structure are evident in Table 1. The number of private facilities is larger than public ones in all countries except DRC. Pharmacies are generally private in all countries. In all countries, public facilities are mostly made up of health posts, health centres and medical centres. The overall number of facilities by type is generally stable across quarters for each country.

Of these facilities, the vast majority offers at least one modern method. For example, 86.9% of all public SDPs and 83.0% of all private SDPs in quarter 1 in Burkina Faso offer at least one modern method. In some settings, however, there is variation across SDP facility types in the percentage offering one modern method: relatively few private hospitals or health clinics/centres in India offer at least one method, but the majority of private pharmacies and drugstores offer at least one method. Similarly, most private pharmacies offer at least one method in Burkina Faso but smaller percentages of medical centres/health centres/health clinics do in all quarters.

### Stockouts and consumption of contraceptive methods

**Figure 1:**
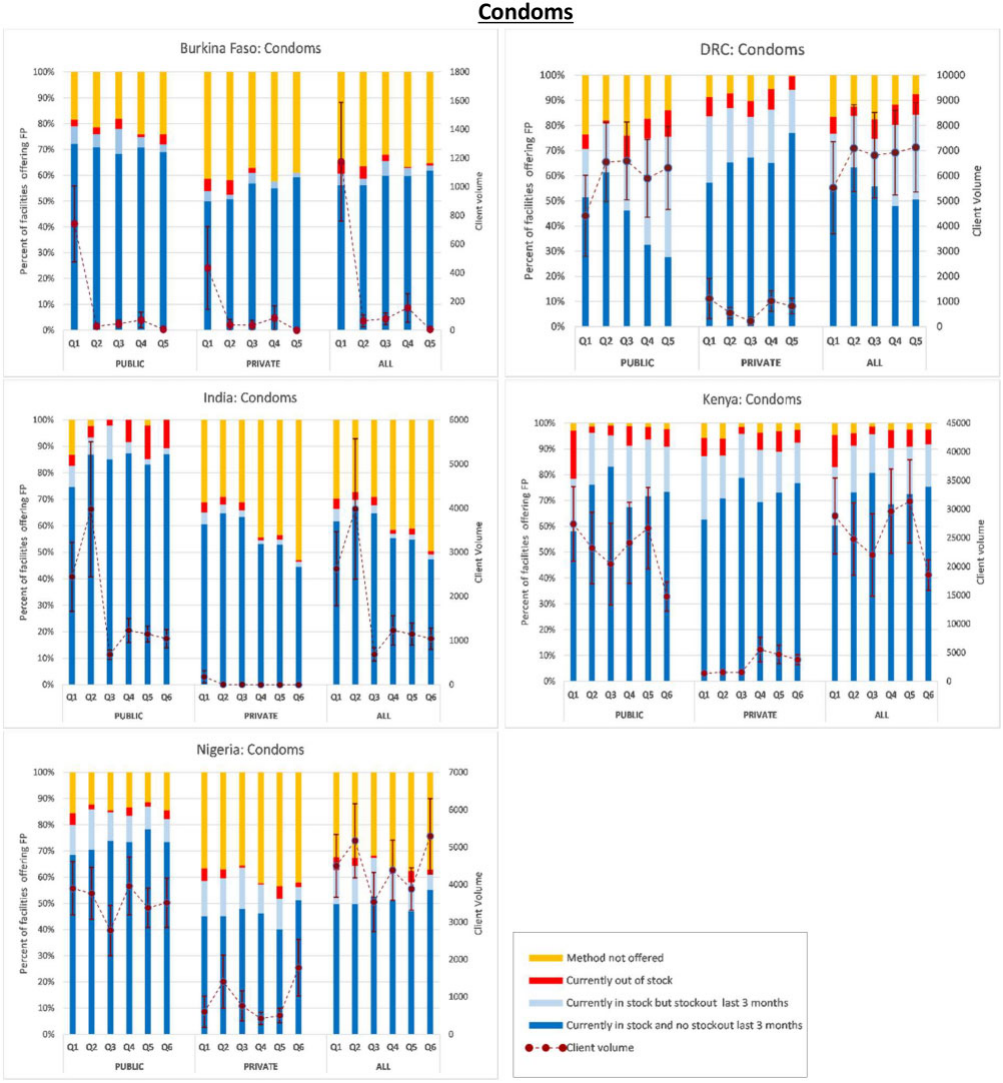
Contraceptive method availability and client volume by quarter and country. NB: Client volume is measured as the total number of visits by method in the month preceding the interview. Error bars represent standard errors

**Figure 2:**
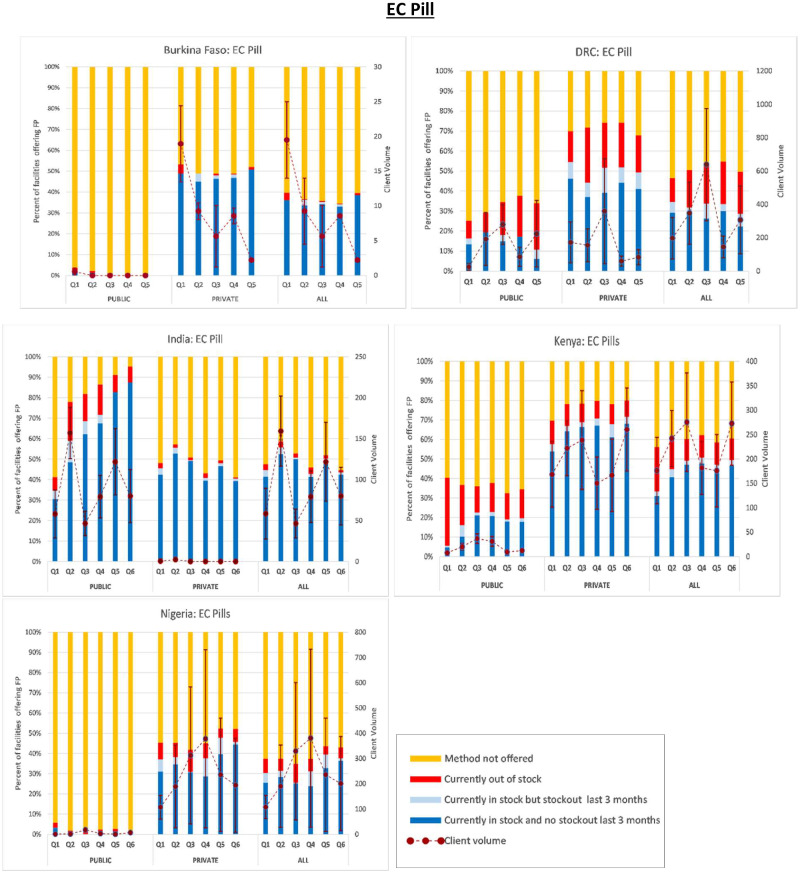
EC pill.

**Figure 3:**
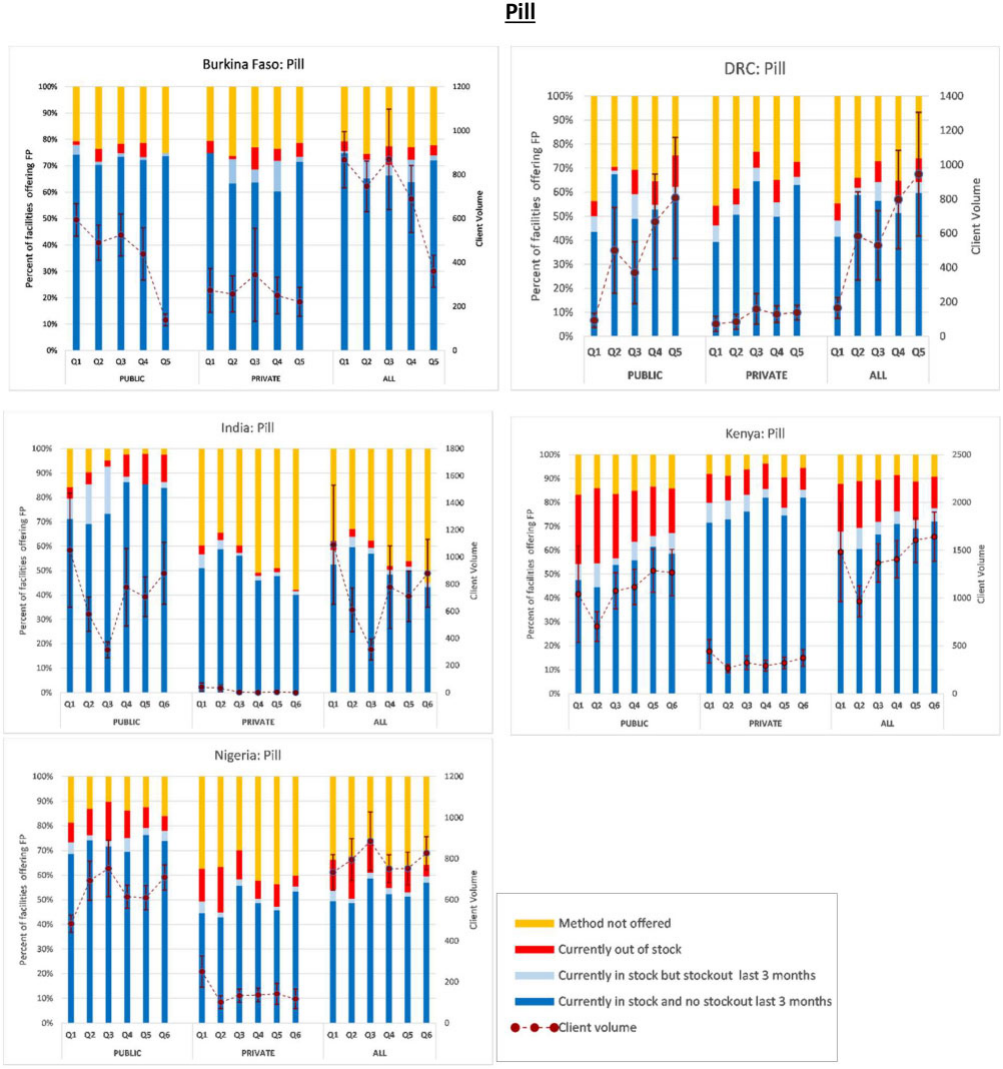
Pill.

**Figure 4:**
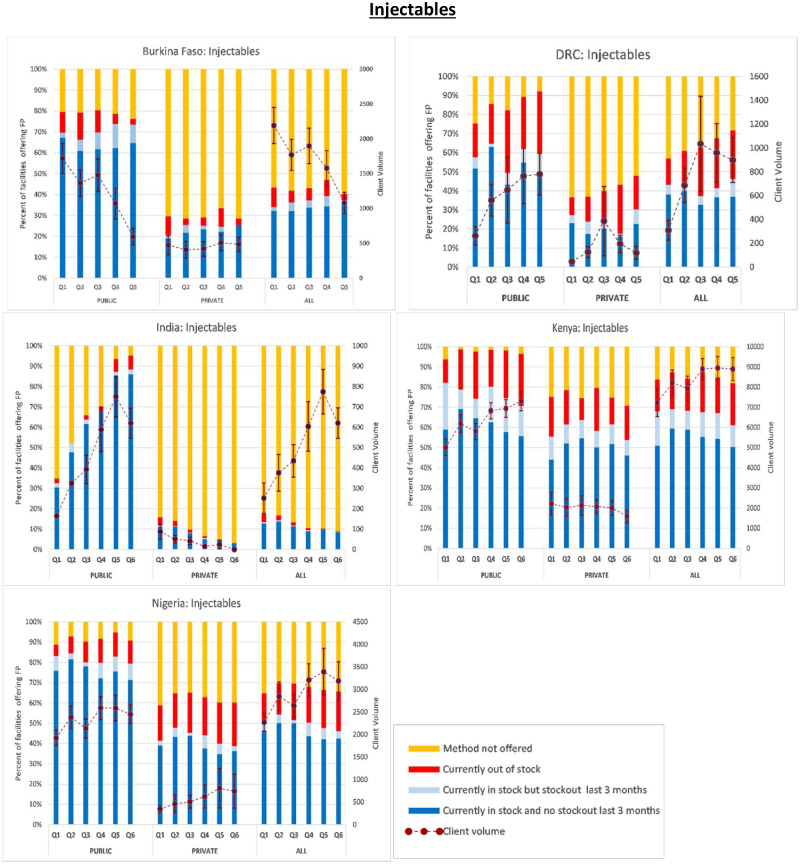
Injectables.

**Figure 5:**
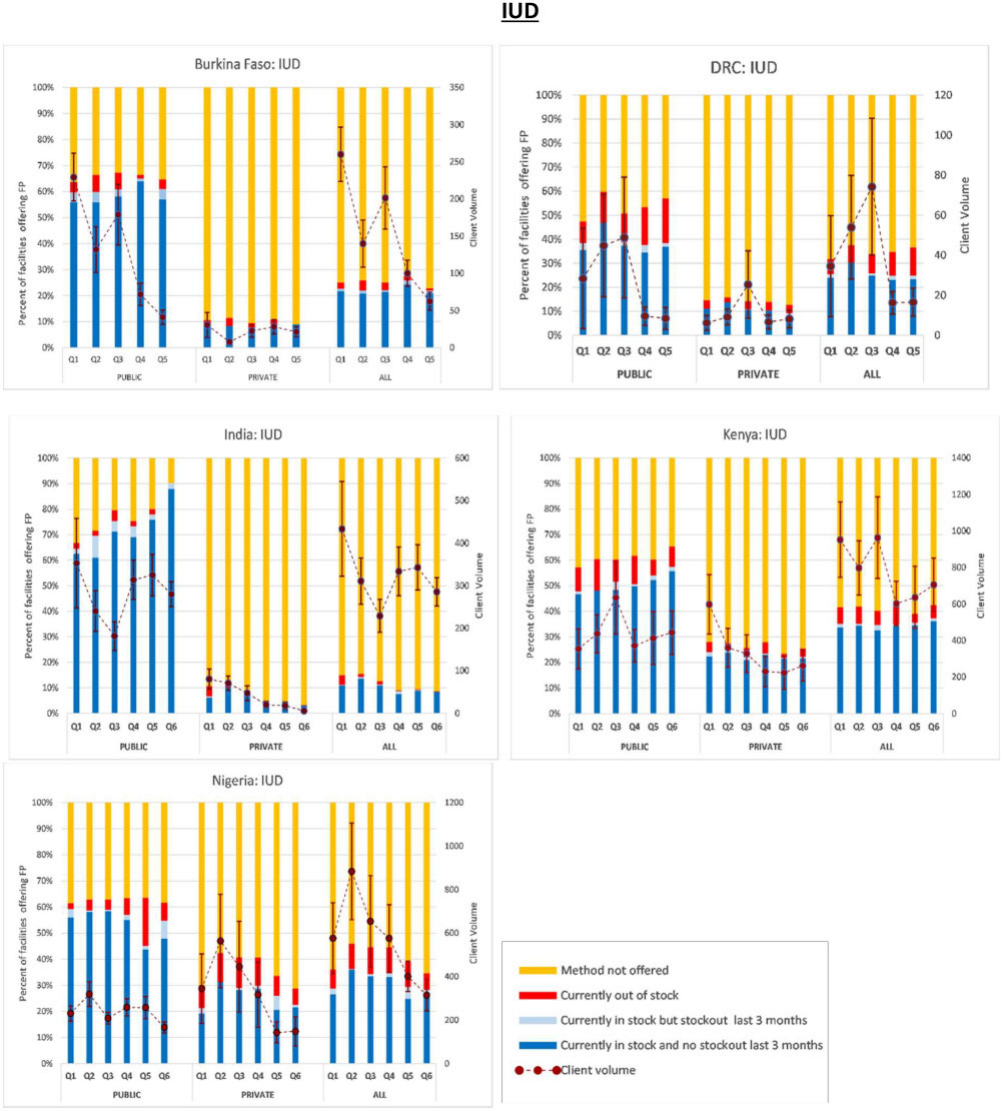
IUD.

**Figure 6:**
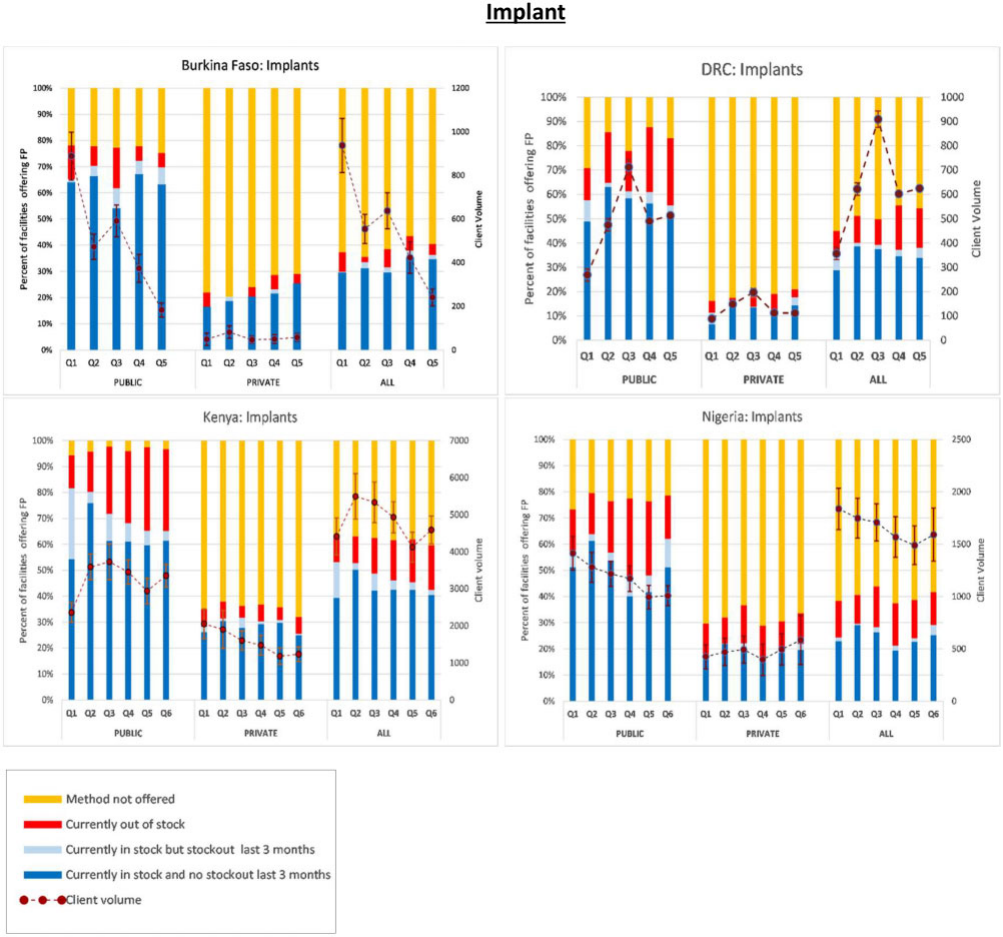
Implant.

Next, in Figures 1–6, we present trends in stockouts for the different types of MCMs (condoms, EC, oral pills, injectables, IUDs and implants), for the public, private and all facilities. In the same figures, we also show the corresponding trends in monthly client volume (in dotted lines). We present this separately for each of the five PMA Agile countries.

#### Condoms

In most countries, condoms were more commonly distributed through the public SDPs instead of the private sector, as shown by higher percentages of public SDPs offering condoms and the higher numbers of monthly client visits purchasing condoms from public SDPs. The average percentage of public SDPs not offering condoms was 11.2% compared to 24.8% of the private SDPs. Among the public SDPs, the average client volume for condoms was 6 842 monthly visits, ranging from 178 visits in Burkina Faso to 22 775 in Kenya. Among the private SDPs, the average client volume was 2018 monthly visits ranging from 32 visits in India to 3077 in Kenya. Regardless of SDP managing authority, an average of 7 858 monthly visits for condoms were observed across all countries ranging from 296 in Burkina Faso to 25 852 in Kenya.

The average SDP stockout rate for condoms was 4.2% across all countries and it ranged from 2.5% in Burkina Faso and India to 6.8% in the DRC. Among the surveyed public SDPs, the average condom stockout rate was 5.3% ranging from 2.5% in Nigeria to 7.3% in Kenya. The average condom stockout rate among the private SDPs was 4.1% ranging from 2.2% in India to 6.6% in the DRC. Furthermore, an average of 12.5% of all the SDPs across all countries had experienced a condom stockout within the 3 months preceding the survey. This percentage varied from 2.8% in India to 25.6% in the DRC.

#### Emergency contraception

We found that the private sector was generally the main distributor of emergency contraceptive pills among the Sub-Saharan African (SSA) countries. In fact, the distribution was conducted almost exclusively through the private sector in Burkina Faso and Nigeria. Among the private facilities with FP, the proportion that did not offer emergency contraception over the study period fluctuated around 28.4% for the DRC, 22.6% for Kenya with that number exceeding 50% for both Burkina Faso and Nigeria. Compared to the other African countries in the study, Burkina Faso had the lowest volume of clients (9 monthly visits on average) purchasing EC with the number progressively decreasing during the study period. In contrast, there were 327, 241 and 221 monthly client visits on average, in the DRC, Nigeria and Kenya, respectively. Across all the African countries, the trends for monthly client visits remained largely highly variable.

Contrary to African countries, the provision of EC in Indian sites was largely via the public sector. The proportion of government-owned SDPs that had EC in stock on the day of the survey increased from 30.6% to 87.4%. The percentage of SDPs experiencing a stockout within the 3 months preceding the survey decreased from 3.1% to 1.1%. This positive trend was further strengthened by the almost 60% reduction in SDPs experiencing a stock out in EC between Q2 and Q6. The steady gains and stability observed in method provision within the public sector were not accompanied by similar increases in client visits for EC. Client visits for EC through the private sector in Indian sites were negligible.

#### Oral pills

Similar to EC, the distribution of oral pills in India was higher in the public sector compared to the private sector. Over the study period, the proportion of public SDPs that did not offer the method in India decreased from 14.8% to 2.3%. This trend was reversed within the private sector with the percentage of SDPs not offering pills increasing from 39.0% to 57.5%. The average number of monthly visits for pills was 718 among the public SDPs compared to only 13 among the private SDPs.

Similarly, in SSA countries, the number of clients obtaining pills was generally higher among public SDPs compared to private SDPs. The ratio of the average monthly client volume among public SDPs to that among private SDPs ranged from 2.2 in Burkina Faso to 4.4 in Nigeria. Compared to other African geographies, Burkina Faso generally experienced lower stockouts of oral pills with an average stockout rate of 3.8% over the study period. In contrast, Kenya experienced an overall average stockout rate of 17% in oral pills with that number approximating 25% among public SDPs. The overall average stockout rate in the DRC was 19.7% (21.2% among public SDPs; 17.8% private SDPs). In Nigeria, the stockout rate for oral pills varied from quarter to quarter at an overall average of 10.6% across surveyed SDPs (10.1% among public SDPs; 10.8% private SDPs).

With the exception of Burkina Faso, where the percentage of SDPs not offering oral pills increased slightly from 20.7% to 22.2% over the study period, the availability of oral pills generally improved among SSA countries. In the DRC, the average percentage of SDPs that did not offer oral pills was 29%, decreasing from 40% to 21%; a 47.5% decrease (49.5% decrease among public SDPs compared to 45.7% decrease among private SDPs). In Kenya, the percentage of SDPs not offering oral contraceptives decreased by 25% over the study period to an average of approximately only 10%. Only 7% of the private SDPs reported that they do not offer the method compared to 15% of the public SDPs. An average of only 14% of the private Nigerian SDPs compared to 38% of the public SDPs did not offer the method, the overall average being 34%.

#### Injectables

Across all geographies, public SDPs generally offered injectables at higher rates than private SDPs. While only an average of 15.8% of the public SDPs did not offer injectables during the study period, this figure was 56.5% among the private SDPs. The combined average was 45.6%. With the exception of Burkina Faso, net improvements were generally observed in the average number of monthly client visits. The most remarkable improvements were noted among public SDPs in India where the number of client visits for injectables increased by more than 3.5-fold over the first five quarters with an associated 93% decrease in the percentage of SDPs not offering the method. Despite these gains, however, the overall availability of injectables remains low in India due to the low rates of injectable availability among private SDPs. On average, 91% of the private SDPs in India did not offer injectables compared to 31.4% among public SDPs and the combined average of 87.2%.

Across all SDPs, the average stockout rate in injectable provision was 13.0% and it varied considerably across geographies ranging from 1.6% in India to 21.6% in the DRC over the study period. The average stockout rate for the public SDPs across all geographies was 13.6% compared to the average stockout rate of 11.9% among private SDPs. Roughly, 5.1% of all SDPs reported a stockout in injectables within the previous 3 months. There was geographic variation in this rate ranging from 0.4% in India to 11.9% in Kenya. Of all public SDPs, 7.4% experienced a stockout during the 3-month period preceding the interview compared to 4.0% of the private SDPs.

#### Intrauterine devices

Compared to the short-acting methods described above, IUDs were generally offered at lower levels across the different geographies. Across all geographies, the percentage of SDPs not offering IUDs was 69.2% and it ranged from 58.8% in Nigeria to 88.4% in India. It was also notable that, across the board, the public sector offered IUDs at considerably higher levels compared to the private sector and was furthermore likely to have the method in-stock over a 3-month period. The average percentage of public SDPs not offering IUDs was 36.0% compared to 81.2% of the private SDPs. Nonetheless, with the exception of Nigeria, IUD stockouts were more common among public SDPs. Of the public SDPs that offered IUDs, the average stockout rate was 7.3% compared to 3.7% among the private SDPs. Across all countries, the combined average stockout rate was 5.4%. On average, 1.1% of the IUD-providing SDPs reported a stockout during the 3-month period preceding the interview. This figure was 2.5% among public SDPs compared to 0.7% among the private SDPs.

Trends in the client visits for the purchase of IUDs were centred at an average of approximately 372 monthly visits. It varied across countries ranging from an average of 39 monthly visits in the DRC to 776 monthly visits in Kenya.

#### Implants

The average percentage of SDPs not offering implants over the study period was 52.0%, ranging from 38.1% in Kenya to 61.0% in Burkina Faso. In all cases, the percentage of public SDPs offering implants was greater than private SDPs. Overall, only 17.1% of the public SDPs did not offer implants compared to 72.4% of the private SDPs. This contrast was starkest in Kenya where a quarterly average of only 3.7% of the public SDPs did not offer implants compared to 64.3% in the private sector (combined average being 38.1%). Among the African countries, the overall average stockout rate was 11.5% ranging from 5.2% in Burkina Faso to 14% in both Kenya and Nigeria. The DRC had an average implant stockout rate of 13.1%. The stockout rates were higher among the public SDPs and varied more across quarters relative to the private SDPs. Among the public SDPs, the average stockout rate was 18.9% compared to 6.6% observed among the private SDPs. Furthermore, whereas the percentage of public SDPs that experienced a stockout in implants 3 months prior to the interview was 6.3%, this number was only 1.3% among the private SDPs. The average number of monthly client visits was 1 913 monthly visits and ranged from 559 visits in Burkina Faso to 4 815 in Kenya.

### Couple-years of protection of contraceptives

CYP data for the different countries are shown in Table 2. Of all the MCMs considered in this study, implants on average accounted for the most CYPs among the public SDPs. With the exception of India where implants are currently not offered, implants accounted for 58.9% of the CYPs provided by the public SDPs across all countries ranging from 55.2% in Burkina Faso to 61.5% in the DRC. This trend did not hold among the private SDPs.

**Table 2:** Quarterly changes in couple years of protection (CYP) by country and method

	PUBLIC												PRIVATE											
Country name^a^	Quarters												Quarters											
	Q1		Q2		Q3		Q4		Q5		Q6		Q1		Q2		Q3		Q4		Q5		Q6	
	Total CYP	%	Total CYP	%	Total CYP	%	Total CYP	%	Total CYP	%	Total CYP	%	Total CYP	%	Total CYP	%	Total CYP	%	Total CYP	%	Total CYP	%	Total CYP	%
Burkina Faso	***N* = 34**		***N* = 32**		***N* = 34**		***N* = 29**		***N* = 15**				***N* = 82**		***N* = 73**		***N* = 75**		***N* = 73**		***N* = 79**			
Condoms	48.4	0.8	4.1	0.1	3.0	0.1	4.3	0.3	2.1	0.2			80.8	9.8	83.9	9.9	70.6	9.4	4.3	0.3	74.9	7.9		
Pill	240.3	4.2	200.6	5.4	200.7	4.8	122.8	7.4	64.2	5.2			229.8	27.9	293.8	34.7	221.7	29.7	122.8	7.4	209.0	22.1		
Injectable	652.1	11.4	501.1	13.6	516.4	12.4	274.0	16.5	202.7	16.6			131.4	16.0	114.9	13.6	117.0	15.6	274.0	16.5	131.6	13.9		
EC pill	0.0	0.0	0.0	0.0	0.0	0.0	0.0	0.0	0.0	0.0			93.5	11.4	104.4	12.3	75.1	10.0	0.0	0.0	151.7	16		
IUD	1545.1	26.9	962.3	26.1	1184.5	28.4	330.3	19.8	293.9	24.0			141.2	17.2	36.3	4.3	122.4	16.4	330.3	19.8	230.0	24.3		
Implant	3257.8	56.7	2016.8	54.7	2268.8	54.4	934.3	56.1	661.5	54.0			145.5	17.7	214.5	25.3	141.0	18.9	934.3	56.1	149.0	15.7		
Total	**5743.7**	100	**3684.8**	100	**4173.3**	**100**	**1665.6**	**100**	**1224.4**	**100**			**822.2**	100	**847.9**	100	**747.8**	**100**	**1666**	**100**	**946.2**	**100**		
DRC	***N* = 33**		***N* = 58**		***N* = 54**		***N* = 66**		***N* = 70**				***N* = 57**		***N* = 70**		***N* = 61**		***N* = 67**		***N* = 65**			
Condoms	296.8	20.5	392.9	18.9	379.6	13.9	339.0	17.0	378.7	17.4			127.5	12.3	103.6	6.3	100.6	6.9	158.3	3.1	138.9	4.1		
Pill	26.0	1.8	140.1	6.8	107.6	3.9	187.0	9.4	226.5	10.4			528.6	51.1	981.7	59.5	529.2	36.3	1199.0	23.5	773.7	22.7		
Injectable	83.4	5.7	140.2	6.8	167.5	6.1	191.1	9.6	197.2	9.1			34.6	3.4	71.2	4.3	136.0	9.3	84.5	1.7	68.2	2.0		
EC pill	4.1	0.3	9.6	0.5	76.9	2.8	4.3	0.2	11.2	0.5			92.6	9.0	83.0	5.0	84.0	5.8	92.1	1.8	168.9	5.0		
IUD	130.6	9.0	206.1	9.9	224.5	8.2	44.2	2.2	38.6	1.8			28.5	2.8	41.9	2.5	116.4	8.0	3280.7	64.3	994.1	29.2		
Implant	910.3	62.7	1185.8	57.2	1783.5	65.1	1225.0	61.5	1321.8	60.8			221.8	21.5	369.3	22.4	492.5	33.8	284.3	5.6	1265.8	37.1		
Total	**1451.2**	100	**2074.6**	100	**2739.6**	**100**	**1990.5**	**100**	**2174.0**	**100**			**1033.5**	100	**1650.7**	100	**1458.5**	**100**	**5098.8**	**100**	**3409.5**	**100**		
India	***N* = 24**		***N* = 22**		***N* = 20**		***N* = 19**		***N* = 20**		***N* = 17**		***N* = 205**		***N* = 246**		***N* = 190**		***N* = 213**		***N* = 227**		***N* = 193**	
Condoms	164.0	12.0	238.5	20.8	41.0	7.3	73.8	4.8	69.6	5.6	62.6	4.2	231.1	14.3	377.7	27.0	336.6	26.3	288.7	8.6	305.5	26.0	134.7	13.7
Pill	303.4	22.1	162.4	14.2	89.3	15.9	217.6	14.2	197.9	16.0	246.4	16.4	319.9	19.8	297.8	21.3	240.7	18.8	117.5	3.5	235.0	20.0	173.0	17.6
Injectable	41.3	3.0	76.2	6.7	90.0	16.0	136.1	8.9	150.7	12.1	30.0	2.0	49.3	3.1	40.0	2.9	30.5	2.4	508.3	15.1	37.9	3.2	40.6	4.1
EC pill	3.3	0.2	7.8	0.7	2.3	0.4	4.0	0.3	6.1	0.5	4.0	0.3	66.9	4.1	79.5	5.7	70.0	5.5	441.5	13.2	101.1	8.6	91.9	9.3
IUD	859.7	62.7	659.6	57.6	339.0	60.4	1102.6	71.9	816.0	65.8	1156.9	77.1	946.2	58.6	602.1	43.1	603.5	47.1	2000.5	59.6	496.3	42.2	544.2	55.3
Total	**1371.8**	100	**1144.6**	100	**561.6**	100	**1534.0**	100	**1240.3**	100	**1499.9**	100	**1613.4**	100	**1397.0**	100	**1281.4**	100	**3356.4**	100	**1175.8**	100	**984.4**	100
Kenya	***N* = 232**		***N* = 223**		***N* = 220**		***N* = 220**		***N* = 218**		***N* = 224**		***N* = 265**		***N* = 283**		***N* = 293**		***N* = 300**		***N* = 292**		***N* = 300**	
Condoms	1647.8	15.0	1393.7	9.8	1229.6	7.9	1447.7	10.3	1601.4	11.9	887.4	6.4	584.5	5.3	388.7	3.4	587.4	6.0	730.0	7.9	606.2	7.9	579.1	7.2
Pill	291.9	2.7	203.5	1.4	306.4	2.0	318.3	2.3	363.9	2.7	363.6	2.6	978.6	8.9	668.8	5.9	813.3	8.3	852.8	9.3	599.3	7.8	827.9	10.3
Injectable	1260.9	11.5	1558.3	10.9	1465.0	9.5	1724.9	12.3	1753.7	13.1	1861.2	13.4	1565.6	14.2	1465.4	12.9	1708.7	17.5	1674.6	18.2	1533.4	19.9	1502.6	18.7
EC pill	0.4	0.0	1.1	0.0	1.8	0.0	1.6	0.0	0.5	0.0	0.6	0.0	231.3	2.1	703.4	6.2	947.1	9.7	826.2	9.0	687.2	8.9	635.9	7.9
IUD	1534.1	14.0	1961.4	13.8	2922.8	18.9	1769.2	12.6	2121.1	15.8	2035.5	14.6	2193.7	19.9	3259.6	28.7	1575.0	16.1	1013.8	11.0	1041.0	13.5	1177.6	14.6
Implant	6221.0	56.8	9136.3	64.1	9553.3	61.7	8755.0	62.5	7591.0	56.5	8768.0	63.0	5472.0	49.6	4860.3	42.8	4159.8	42.5	4104.0	44.6	3230.5	42.0	3326.8	41.3
Total	**10956.1**	100	**14254.2**	100	**15479**	100	**14016**	100	**13432**	100	**13916**	100	**11025.7**	100	**11346.2**	100	**9791.3**	100	**9201.5**	100	**7697.6**	100	**8049.9**	100
Nigeria	***N* = 79**		***N* = 87**		***N* = 88**		***N* = 88**		***N* = 82**		***N* = 79**		***N* = 270**		***N* = 325**		***N* = 342**		***N* = 338**		***N* = 302**		***N* = 285**	
Condoms	271.0	4.7	232.1	4.0	174.9	3.4	248.6	4.1	209.1	4.0	243.0	4.4	235.2	4.7	230.1	4.1	170.1	3.7	187.3	5.0	205.8	5.3	281.4	7.8
Pill	152.7	2.7	198.5	3.4	222.6	4.3	181.8	3.0	194.6	3.7	238.7	4.3	328.5	6.6	301.4	5.4	302.8	6.5	281.1	7.5	349.1	8.9	299.8	8.4
Injectable	499.7	8.7	606.1	10.5	549.8	10.7	677.6	11.3	691.6	13.1	724.7	13.1	287.7	5.8	391.0	7.0	409.8	8.9	360.2	9.6	522.4	13.4	303.6	8.5
EC pill	0.0	0.0	0.1	0.0	1.0	0.0	0.4	0.0	0.0	0.0	0.8	0.0	153.9	3.1	206.9	3.7	163.0	3.5	248.1	6.6	511.0	13.1	319.2	8.9
IUD	1103.1	19.2	1491.8	25.8	1013.8	19.7	1779.3	29.6	1299.5	24.6	994.1	17.9	2499.2	50.3	2984.5	53.1	2261.4	48.9	1587.0	42.3	845.5	21.7	753.9	21.0
Implant	3707.8	64.7	3246.5	56.2	3184.8	61.9	3115.5	51.9	2883.3	54.6	3337.3	60.3	1460.3	29.4	1504.3	26.8	1320.8	28.5	1085.3	28.9	1470.5	37.7	1627.0	45.4
Total	**5734.3**	100	**5775.0**	100	**5146.8**	100	**6003.1**	100	**5278.1**	100	**5538.5**	100	**4964.7**	100	**5618.2**	100	**4627.8**	100	**3748.8**	100	**3904.3**	100	**3584.9**	100

*N* represents the total weighted number of SDPs contributing to CYP units

Couple years of protection (CYP) is the estimated protection provided by contraceptive methods during a 1-year period, based upon the volume of all contraceptives sold or distributed free of charge to clients during that period (USAID, 2019). the bold values differentiate country totals from other values for the individual methods

Consistent with the lower availability rates and client volumes observed among public SDPs offering emergency contraception, we found that this method did not provide many CYPs relative to other MCMs offered by the public SDPs.

In Burkina Faso, an average of 4 146 CYPs was provided each quarter, of which 3 298 were provided by public SDPs with the remainder being provided by the private SDPs. The combination of IUDs and implants accounted for 80.2% of the CYPs provided by public SDPs and 33.8% of those provided by private SDPs. In the DRC, public SDPs provided an average of 2 086 CYPs each quarter compared to 2 530 among the private SDPs. Oral pills contributed the highest amount of CYPs in the private sector. They accounted for 38.6% of the total CYPs in the private sector. IUDs and implants together accounted for 45.5% of the CYPs provided by the private SDPs. In India, public SDPs provided an average of 1 225 CYPs whereas the private SDPs contributed 1 635 CYPs. Among the MCMs tracked in this study, IUDs provided the highest amount of CYPs against unintended pregnancies. This was consistent over time among both public and private SDPs. Relative to other MCMs, injectables and emergency contraceptive pills did not seem to provide high levels of protection in India. We found that the public SDPs in Kenya provided 13 676 CYPs against unintended pregnancies on average compared to the 9 519 observed in the private sector. Implants provided the most protection across all SDPs regardless of public–private ownership. Similar to the public SDPs in Kenya, those in Nigeria provided a higher amount of protection (5 579 CYPs) compared to their private counterparts (4 408 CYPS). Together, implants and IUDs generally provided the most couple-years of protection across both public and private SDPs.

## Discussion

Modern contraceptive use is a product of both supply of and demand for contraception. While the latter has been examined extensively, the former has been studied less often, largely due to data limitations. In this research, we use locally representative data to measure trends in the family planning supply environment in urban or suburban areas of Burkina Faso, DRC, India, Kenya and Nigeria. Specifically, we describe trends in stockouts, method availability and consumption of specific contraceptive methods.

Although each setting has its own unique FP supply profile, some of our results are consistent across settings. We find that public sector facilities are more likely to have a comprehensive mix of short- and long-acting contraceptive methods, compared to private sector facilities, similar to results from other studies (Hutchinson *et al.*, 2011; Kakoko *et al.*, 2012). There are, however, a few exceptions to this pattern, such as EC in SSA countries and oral pills in Kenyan sites. Indeed, previous work by others has shown that private sector SDPs are the major outlets for EC in a range of SSA countries (Maharaj and Rogan, 2008; Mané *et al.*, 2015; Hernandez *et al.*, 2018) and for oral pills in Kenyan sites (Corroon *et al.*, 2016). Furthermore, it is worthwhile to point out that, for some SSA countries such as Kenya, the distribution of EC in public facilities is usually provided only to victims of sexual assault or rape whereas it is readily available over the counter without a doctor prescription in private pharmacies (Thompson *et al.*, 2018). The exact difference between public and private facilities in contraceptive availability differs by country and method. For example, the levels of condom availability are similar between public and private facilities in Kenya, but the difference is larger for IUD and implant availability.

Analysis of MCM stocks by facility type confirmed previous findings by others that while primary care facilities are important outlets for contraceptives in the public sector, pharmacies and drug shops dominate the private sector. Pharmacies and drug shops have an important role to play in achieving FP2020 objectives particularly in SSA countries where they serve as crucial outlets for addressing the unmet need in short-acting contraceptives for vulnerable and hard-to-reach populations (Corroon *et al.*, 2016; Riley *et al.*, 2018). Recent evidence points to the popularity of drug shops, in particular, as preferred SDPs for young women and unmarried women (High-Impact Practices in Family Planning, 2013; Corroon *et al.*, 2016; Weinberger and Callahan, 2017), though their potential likely remains under-leveraged.

In general, we noted that the patterns of stock supply and client volume corroborated findings from previous studies examining client-level data in contraceptive use. For example, the high client volume and method availability observed across the sites in Kenya relative to other SSA countries is consistent with a previous study showing that Kenya had the highest modern contraceptive rate among the countries in the study (Ahmed *et al.*, 2019).

Consistent with other studies, our findings showed limited availability for long-acting reversible contraceptives (LARCs), including IUDs and implants, with great variability between countries (Grindlay *et al.*, 2016; Thanel *et al.*, 2018). The findings further showed that LARCs were generally more readily available and distributed through the public sector as compared to the private sector. LARCs are highly effective forms of reversible birth control as evidenced by the large proportion of CYPs they provided in our study despite the consistently high levels of stockouts and lower numbers of client visits that they were subject to. Compared to short-acting methods, they have higher upfront costs but are more cost-effective over time, generally well-tolerated by women and thus less likely to be discontinued (World Health Organization, 2012; Diedrich *et al.*, 2015; Thanel *et al.*, 2018; Römer and Linsberger, 2009). Nonetheless, the requirements for service readiness for LARC delivery are decidedly more complex than those for short-acting methods. For instance, service readiness for implant and IUD insertion typically requires the availability of the contraceptive commodity, appropriately trained and credentialed providers and the necessary equipment for insertions. It is thus likely that the low levels of LARC availability observed across countries can be attributed to the limited capacity of country health systems to sustain adequate levels of service readiness for LARCs. With the aim of addressing the shortcomings related to service readiness, some countries, including Nigeria, have made commitments to increase FP program funding while pursuing task-shifting policies geared towards expanding service delivery for LARCs (Scoggins and Bremner, 2020). Nonetheless, our findings suggest that further action is needed from governments, FP program managers and international donors to leverage underutilized private sector primary care providers and continue striving towards improved access to LARCs.

Based on client volume data for LARCs, we observed that implants were consistently distributed at higher levels than IUDs in SSA. This is consistent with previous studies that have shown higher levels of implant use relative to IUDs in several African countries (Duvall *et al.*, 2014; Tsui *et al.*, 2017). While implants possess relative advantages and attributes that users like about them as previously discussed by others (Ortayli, 2002; Jacobstein and Stanley, 2013), it is worthwhile to also note that the introduction of implants in SSA benefitted from considerable international assistance to expand access to the method and manage supply chains (Duvall *et al.*, 2014; Tsui *et al.*, 2017).

Total CYPs in India appeared to be lower than those in SSA countries. This was likely due to the lack of contribution of implants to total CYPs as the method is currently not offered in India. Though implants are offered in the DRC, the total CYPs provided were still low compared to other African geographies likely due to the low client volume for IUDs. These findings not only highlight the importance of ensuring the availability of LARC choice for maximum protection against unintended pregnancies but also the value of examinations of country-specific method mix when attempting cross-country comparisons. As others have previously reported, trends in method mix may vary considerably across and within geographies thereby raising programmatic issues that need to be addressed in order to address client needs (Bertrand *et al.*, 2020).

Our goal in this research is primarily descriptive; we do not seek to explain why stockouts occur or why certain methods are consumed more than others, as doing so would require the measurement of environmental and supply factors that were out of the scope of PMA Agile. For example, we understand from correspondence with PMA Agile personnel that declines in stock of MCMs and in client visits for MCMs in Burkina Faso were due to a public sector strike that took place between June and November of 2019. Additionally, the Indian MoH launched the ‘Antara Programme’ in 2017 with the aim of providing injectables free of charge in public facilities (Ministry of Health and Family Welfare, 2017b). Though this potentially explains the remarkable increase in injectable availability and consumption in the public sector in India, we do not capture information on such programmatic changes, potential disruptions to service delivery or other similar environmental impacts on stockouts.

Although PMA Agile did not collect information on the reason for stockouts, the PMA Core project (PMA, 2020) recently did so in four geographies that overlap with the countries chosen here: Kenya, Burkina Faso, DRC and Nigeria. Across all countries and methods, the most common reason for stockouts is that the facility claims to have ordered the method but did not receive the requested shipment. The delays in making contraceptives available in India are likely due to weak monitoring of the contraceptive method supply and consumption at all levels of the health system (Ministry of Health and Family Welfare, 2017a). In response, the Indian government recently rolled out the Family Planning Logistics Management Information System (FPLMIS) (Ministry of Health and Family Welfare, 2017a). The FPLMIS is expected to empower state-level program managers, facility-level stock managers as well as community health workers manage stocks of contraceptive commodities more effectively.

Although a detailed examination of the relationship between contraceptive procurement, supply chain management and stockouts was beyond the scope of this research, our findings nonetheless have the potential to inform ongoing debates about the merits of different contraceptive distribution models vis-à-vis stockouts. For example, whereas Burkina Faso and DRC use a ‘pull system’ in which procurement is decentralized and multiple low-level actors place contraceptive orders from central hubs based on their forecasted needs (PATH, 2017; Babazadeh *et al.*, 2018), Nigeria uses a variation of the push system in which the central hubs are responsible for resupplying SDPs with contraceptives (USAID | DELIVER PROJECT, 2014) with Kenya using a combination of the two systems (Eliya Msiyaphazi Zulu *et al.*, 2012). Yet, it was not entirely clear from our findings whether contraceptive stockouts vary based on the distribution model employed, considering the differences in contraceptive consumption across countries. Given our observations that stockouts vary based on geographic setting, sector and contraceptive method, we recommend that country stakeholders adopt context-specific and problem-based approaches to address procurement challenges for individual methods. Considering the rapid fluctuations observed in both contraceptive consumption and stockout rates, the approaches will need to be flexible and cost-effective in order to sustainably address unmet need.

On the basis of the PMA Core observation that failure to receive requested contraceptive orders was the most common cause of stockouts among SDPs in African geographies, it is probable that countries may benefit from the Informed Push Model (IPM), which achieved dramatic reductions in stockouts in Senegal largely by transferring the responsibility of order placement and delivery from SDPs to an external professional logistician (Daff *et al.*, 2014) The model’s ability to couple information collection and product distribution by dedicated professionals enhances supply chain performance ultimately strengthening the health system’s ability to respond to the healthcare needs of the population. Furthermore, IPM avoids the requirement for facilities to pay for contraceptive supplies upfront, a situation which may exacerbate supply chain issues (Hasselback *et al.*, 2017). As others have indicated, upfront payments may drive some facilities to use their working capital thus delaying the replenishment of funds until after clients have purchased contraceptive commodities (Hasselback *et al.*, 2017). The funding delays may in turn result in cash flow problems for some facilities or encourage the diversion of the remaining capital towards more profitable non-contraceptive commodities. Given the success of IPM in addressing these issues, we therefore recommend country-specific analyses comparing costs associated with current distribution channels for individual methods to the costs associated with alternative distribution models including IPM.

There is limited data on MCM stockouts in low- and middle-income settings, and most available data offer annual averages that do not provide the opportunity to examine short-term trends in stockouts. The quarterly PMA Agile data therefore fill an important gap in the literature, by tracking within-year changes in stockouts, CYPs and client volume across five different settings. We also provide a contrast between public and private facilities which greatly increases the representativeness of our findings.

This research also has some limitations. The PMA Agile platform offers an online dashboard displaying trends obtained from the analysis of repeated cross-sectional surveys (PMA Agile, 2020). There may be instances where our results differ slightly from the dashboard results since our analytical dataset contained SDPs that were followed longitudinally across at least two quarters. We also note that in some instances, such as in the public sectors for Burkina Faso and India, the sample size of facilities is small. Furthermore, our analyses combine different types of SDPs which are in turn probably subject to different variation in terms of method availability and client volume. In future studies, we plan to conduct facility-level analyses that address these concerns. Though REs were able to access facility logbooks and record product sales and client visit data, we are unable to comment on the extent to which the data were accurate or complete. With the exception of SDPs in Burkina Faso and public SDPs in DRC, the proportion of facilities contributing data was generally high across countries as evidenced by the number of SDPs contributing to CYP units.

Finally, the survey data were subject to sampling and non-sampling errors.

The majority of research on FP and contraceptive use has focused on demand-side measures, such as fertility preferences, individual-level predictors of modern contraceptive use and characteristics associated with unmet need. In this research, we demonstrate the value of supply-side measures by showing trends in contraceptive supply and stockout over a short period of time. We expect that this information may help monitor progress towards addressing an unmet need and inform cross-country strategies to anticipate, reduce and prevent stockouts.

## Supplementary Material

czaa197_Supp
